# Pembrolizumab-Associated Cardiotoxicity: A Retrospective Analysis of the FDA Adverse Events Reporting System

**DOI:** 10.3390/ph17101372

**Published:** 2024-10-15

**Authors:** Stefan Milutinovic, Predrag Jancic, Vera Jokic, Marija Petrovic, Igor Dumic, Ambar Morales Rodriguez, Nikola Tanasijevic, Dustin Begosh-Mayne, Dragana Stanojevic, Ricardo O. Escarcega, Juan Lopez-Mattei, Xiangkun Cao

**Affiliations:** 1 Internal Medicine Residency Program at Lee Health, Florida State University College of Medicine, Cape Coral, FL 33909, USA; 2 Faculty of Medicine, University of Belgrade, 11000 Belgrade, Serbia; 3 Montefiore New Rochelle Hospital, New Rochelle, NY 10801, USA; 4 Cardiology Fellowship Program, Icahn School of Medicine at Mount Sinai, New York, NY 10029, USA; 5 Department of Hospital Medicine, Mayo Clinic Health System, Eau Claire, WI 54703, USA; 6 NYC Health + Hospitals/North Central Bronx, Bronx, 10467 NY, USA; 7 Clinic for Cardiology, University Clinical Center Nis, 18000 Nis, Serbia; 8 Lee Health Heart Institute, Fort Myers, FL 33908, USA

**Keywords:** pembrolizumab, cardiotoxicity, immune checkpoint inhibitors, FAERS

## Abstract

Background: Immune checkpoint inhibitors (ICIs) have been successfully used in the previous decade for the treatment of a variety of malignancies. Adverse events (AEs) can cause many symptoms, most notably cardiac. We analyzed the frequency of these adverse events, comparing pembrolizumab and other ICIs. Methods: Using the Food and Drug Administration (FDA) adverse event reporting database (FAERS), we searched for all adverse events of interest reported for every ICI included in this study. After obtaining the data, we conducted a disproportionality analysis using the reporting odds ratio (ROR) and the information component (IC). Results: A total of 6719 ICI-related cardiac adverse events of interest were reported in the database. Serious outcomes were reported in 100% of the cases, with 34.3% of the cases ending fatally. Compared with all other medications in the database, pembrolizumab use was more frequently associated with myocarditis, pericardial disease, heart failure, and atrial fibrillation. No difference was found in cardiotoxicity between different ICIs. Conclusions: Although infrequent, cardiac AEs in pembrolizumab use are associated with serious outcomes and high mortality. Prospective studies are needed to further research the connection between ICI use and cardiotoxicity.

## 1. Introduction

The immune checkpoint inhibitors (ICIs) are monoclonal antibodies (mAb) that have revolutionized treatment of different malignancies and improved outcomes for cancer patients with advanced malignancies. They target two different pathways: cytotoxic T-lymphocyte-associated antigen 4 (CTLA-4) and programmed cell death protein 1 (PD-1) or its ligand (PD-L1) [1]. Third generation ICIs target lymphocyte activation gene 3 (LAG-3) [2]. ICIs that are currently approved by the FDA are pembrolizumab, nivolumab, cemiplimab, dostarlimab, retifanlimab, toripalimab, and tislelizumab that target PD-1, atezolizumab, avelumab, and durvalumab that target PD-L1, ipilimumab and tremelimumab that target CTLA-4, and relatlimab and nivolumab that target LAG 3/PD-1.

Immunomodulation represents a current state-of-the-art therapeutic strategy in oncology; however, this process is also associated with many side effects, known as immune-related adverse events (irAEs) [3]. Although cardiovascular toxicity is rarely represented in immune-related adverse events, compared with dermatologic, gastrointestinal, hepatic, or endocrine, it can be life-threatening, or in some cases, even fatal. High fatality rates with cardiac immune-related adverse events have been reported in multiple prior post-marketing studies [4,5,6,7,8,9,10].

Anthracyclines and anti-human epidermal growth factor receptor type 2 (HER2)-targeted monoclonal antibodies have well-known cardiotoxicity manifesting as cardiomyopathy, heart failure (HF), systolic or diastolic dysfunction, or reversible decrease in the left ventricular ejection fraction. However, the mechanisms, clinical manifestations, timing, incidence, and management of immune-related cardiac AEs that more commonly present with myocarditis, pericarditis, arrythmias, vasculitis, dyslipidemia, myocardial infarction (MI), or HF [11] are different from those of chemotherapy- or targeted therapy-induced cardiotoxicity. Even though cardiotoxicity is not unique to ICIs, cardio-oncology is an evolving field. Many patients with cancer now live longer, immunotherapies are being granted approval for many types of cancer, and different monoclonal antibodies are used in combination with different other agents, which can increase rates of cardiotoxicity [12,13,14].

The FDA adverse event reporting system (FAERS) contains adverse events reported by manufacturers, healthcare professionals and consumers, as a part of the FDA’s post-marketing safety surveillance program for all marketed drug and therapeutic biologic products [15]. We retrospectively reviewed the FDA adverse events system (FAERS) to assess the incidence of cardiotoxicity linked to pembrolizumab and to compare it with other PD-1/PD-L1/CTLA-4 agents.

## 2. Results

**Figure 1:**
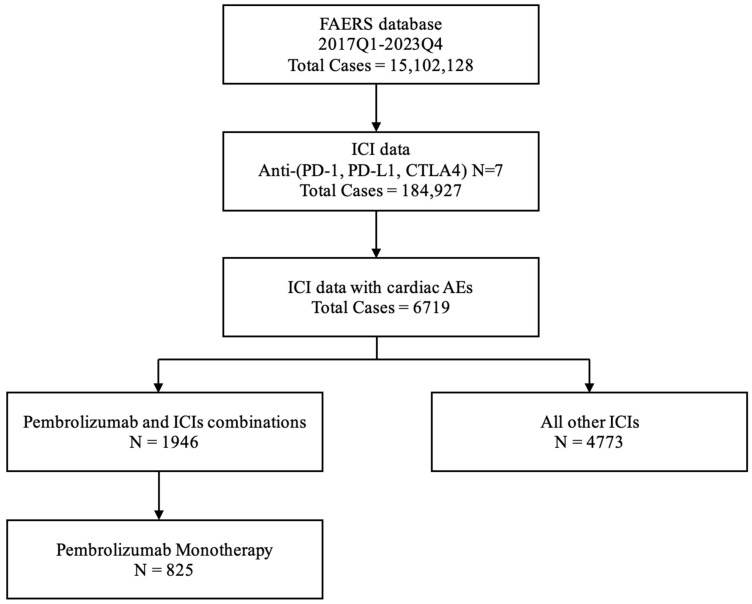
Flow chart showing the analysis process of the study.

Between 1 January 2017 and 31 December 2023, there were a total of 15,102,128 AEs reported in the FAERS database. Of those, 184,927 were due to ICIs, with 6719 (3.6%) being cardiac-related. The total number of cardiac AEs of interest caused by pembrolizumab alone was 825 (17.3% of all pembrolizumab-associated AEs). On the other hand, the total number of cardiac AEs of interest caused by other ICIs was 4773 (Figure 1).

Pembrolizumab-associated cardiac AEs of interest were increasingly reported each year from 2017 to 2023, with 2023 being the most reported year with 139 AEs (16.8%). Among cardiac AEs, gender distribution was not equally represented, with 301 (36.4%) females and 500 (60.6%) males. Among males, myocarditis was the highest reported adverse event (n = 158, 31.60%), compared with atrial fibrillation, which was the lowest reported (n = 68, 13.60%). In 24 (2.9%) reports, the gender was not specified. Serious outcomes were reported in 825 (100%) cases, among which the most common serious outcome was death (n = 283, 34.3%), followed by hospitalization (n = 251, 30.4%), life-threatening events (n = 117, 14.2%), and disability (n = 1, 0.1%). The most reported AE with pembrolizumab alone was myocarditis (n = 276, 33.5%), followed by heart failure (n = 158, 19.2%), pericardial effusion (n = 143, 17.3%), atrial fibrillation (n = 111, 13.5%), myocardial infarction (n = 96, 11.6%), and pericarditis (n = 41, 5%). Death was attributed most often to myocarditis (n = 112, 39.6%), followed by heart failure (n = 67, 23.7%).

Atrial fibrillation associated with pembrolizumab was reported in 111 (13.5%) cases, among whom 61.26% were males. The mean age was 71.58 ± 8.05. All reactions were reported as “serious”, among which death was reported in 24.32%, life-threatening reactions in 13.51%, and hospitalization in 31.53%. Adverse events were reported by the consumers themselves in 16 cases (14.4%) and by healthcare professionals in 94 cases (84.7%), and in 1 (0.9%) case, the reporter was not specified. A total of 38 reports (34.2%) came from the USA, while the rest were from different countries in Europe and Asia. The year 2018 had the highest number of reports, compared with 2020 when only seven reports were sent. Since 2020, there has been a steady increase in the number of cases, reaching 21 cases in the year 2023.

Cardiac failure was attributed to pembrolizumab in 158 (19.2%) reports. Males were predominantly reported (63.52%) with an overall mean age of 71.62 ± 9.69. All reactions were reported as “serious”, with the second highest death rate among adverse events of interest (42.14%). Customers reported adverse events in 10 reports (6.3%), while the rest were reported by healthcare professionals (145, 91.8%). Only three reports had unspecified reporters. The highest number of reports was sent in 2018, with a slow decline after that. In 2023, there were 24 cases reported. Most reports came from Japan (45.5%), followed by the USA with 30 reported cases (19.0%).

Myocardial infarction was reported in 96 cases (11.6%), among whom 71.88% were males. The overall mean age of patients was 70.00 ± 10.55. Similarly, all reports were marked as “serious” and mostly reported by healthcare professionals (76.04%). Death was documented in 46.88% of reports. Most of the events occurred in the USA (37.5%). In the 2017–2023 period, myocardial infarction was consistently reported at a rate of between 11–20 cases per year.

Myocarditis was the most commonly documented adverse event, with a total of 276 (33.5%) reports. Similar to other adverse events, males were predominantly affected (57.25%), with an overall mean age of 69.59 ± 11.55. All adverse events were documented as “serious,” among which 40.58% reported death as an outcome. Healthcare providers were marked as the reporters in 95.3% of reports, while the rest were from consumers (4.7%). The highest number of reports came from the USA (22.8%), France (19.2%), and Japan (14.9%). Interestingly, myocarditis has been increasingly reported over the years, with 32 cases in 2020, 43 cases in 2021, 48 cases in 2022, and 67 cases in 2023.

**Figure 2:**
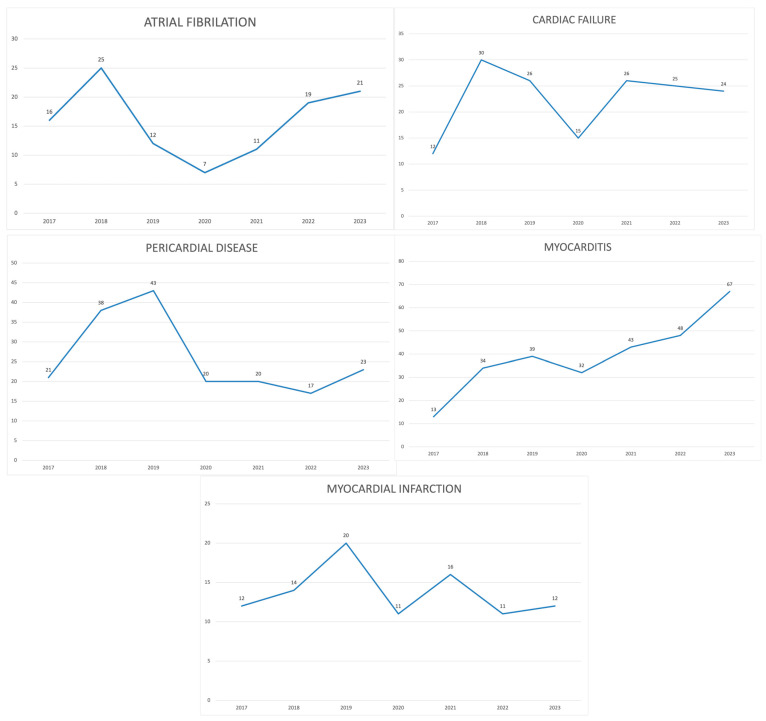
Trend lines for specific cardiotoxicity AEs of interest for 2017–2023, based on the received FDA data.

Pericardial disease (including both pericarditis and pericardial effusion) was present in 172 (20.8%) reports, with a more equal gender distribution (56.52% vs. 43.48% for males and females, respectively) compared with other adverse events. The overall mean age was 63.80 ± 13.22. Most of the reports were sent to the FDA by healthcare providers (98.3%). The USA and Japan sent 54.7% of the reports. The highest number of cases (n = 43) was reported in 2019, with a significant decline after that (Table 1, Figure 2).

According to the disproportionality signal analysis, pembrolizumab use had the strongest statistical association with myocarditis when compared against the database (ROR 33.87, 95% CI 29.98–38.26; IC 4.91, 95% CI 4.71–5.39). Additionally, statistically significant association with pericardial involvement (ROR 6.41, 95% CI 5.54–7.41; IC 2.64, 95% CI 2.39–3.22), heart failure (ROR 1.69, 95% CI 1.44–1.97; IC 0.75, 95% CI: 0.48–1.37), and atrial fibrillation (ROR 1.43, 95% CI 1.18–1.72; IC 0.51, 95% CI 0.19–1.26) was found. Myocardial infarction (ROR 1.22, 95% CI 1.00–1.49; IC 0.28, 95% CI −0.06–1.09) did not meet our criteria for statistical significance based on IC. Lastly, no cases of QT segment prolongation were reported with pembrolizumab (Table 2).

Disproportionality signal analysis, using the ROR to compare pembrolizumab with other ICIs used in monotherapy, found that pembrolizumab did not have a higher statistical association with any of the AEs of interest. Among all ICIs, cardiac failure was most commonly reported with cemiplimab; however, it was not statistically significant based on the reporting odds ratio (ROR 2.05, 95% CI 0.92–4.61). The lowest reporting odds ratio was found with ipilimumab (ROR 0.17, 95% CI 0.07–0.40). Avelumab was the only ICI that was positively associated with atrial fibrillation, although not statistically significant (ROR 1.31, 95% CI 0.72–2.38; IC 0.37, 95% CI −0.66–2.69). Myocardial infarction had higher, but not statistically significant, odds of reporting among all ICIs except with ipilimumab (ROR 0.37, 95% CI 0.15–0.89; IC −1.33, 95% CI −2.89–2.01). Additionally, ipilimumab had less frequent odds of reporting in relation to pericardial disease (ROR 0.17, 95% CI 0.06–0.44; IC −2.42, 95% CI −4.18–1.26). Lastly, myocarditis had a higher reporting odds ratio with cemiplimab, but this was not statistically significant (ROR 1.31, 95% CI 0.54–3.16) (Table 3 and Table 4).

## 3. Discussion

We performed an extensive analysis of pembrolizumab cardiovascular AEs from the FDA pharmacovigilance database. Our analysis showed that reported cardiac AEs related to ICIs have increased over the years, probably reflecting an increase in their use as well as better recognition and reporting. We compared the results with all other drugs in the database, and also with other ICIs. Our analysis showed that cardiac AEs caused by ICIs were infrequent (3.2% by pembrolizumab alone; 3.6% when pembrolizumab was combined with another ICI; 3.6% by the rest of the ICIs). All pembrolizumab-associated cardiac AEs were classified as serious, and the most frequently reported AE caused by pembrolizumab was myocarditis. AEs were more common in men and resulted in death in 34.4% of cases.

**Figure 3:**
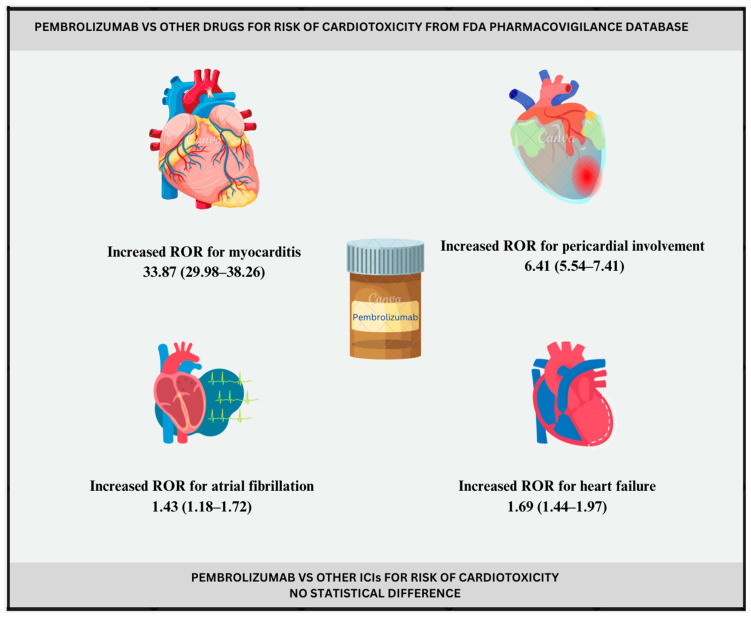
Pembrolizumab and cardiotoxicity; illustration. ICIs-Immune check point inhibitors.

Furthermore, pembrolizumab was found to be more often reported with myocarditis, pericardial disease, heart failure, and atrial fibrillation compared with other drugs in the FAERS database. Among these, the most significant association was found with myocarditis. Conversely, QT prolongation was not reported with pembrolizumab at all. In addition, there was no statistical difference in AE reporting between different immune checkpoint inhibitors (Figure 3).

Over the past decade, pembrolizumab (and other ICIs) has improved mortality and morbidity in cancer patients and become a key component in cancer management [16,17]. However, with increased use of these drugs, irAEs have emerged as an important concern, characterized by excessive T-cell activation, immune tolerance disbalance, and autoimmune response to normal native tissue, including the heart [18].

Immune-checkpoint inhibitors can cause a vast variety of irAEs, with some studies suggesting that up to 90% of treated patients reported some form of adverse event [19]. The most commonly reported adverse events are skin-related, such as a rash or itching, followed by gastrointestinal issues (diarrhea or colitis) [20]. Endocrine dysfunction can range from thyroid and adrenal disorders to even diabetes mellitus [21]. Musculoskeletal and ocular toxicity is also common [19,22]. Rare yet serious irAEs include myocarditis, neurotoxicity, pneumonia, and nephritis [23,24].

Cardiac-related toxicity was underestimated in the pivotal trials but has been increasingly reported in recent years. This underestimation stemmed from a highly diverse clinical presentation and diagnostic challenges surrounding cardiotoxicity, especially the most common presentation, which is myocarditis. The gold standard tests, endomyocardial biopsy and cardiac magnetic resonance, are both expensive and widely unavailable to patients [25,26]. However, troponin may prove to be an inexpensive marker of myocyte damage in myocarditis [27]. Conversely, looking at the curves representing reporting of AEs by year, a significant decrease in the number of cases can be seen starting from 2020 and continuing in the following years. Such a finding can be explained mainly by the start of the COVID-19 pandemic, when reporting of a variety of data might have been skewed in favor of reporting the infection and its complications. Additionally, deaths from the infection could have masked deaths from different etiologies, thus seemingly lowering the likelihood that a certain outcome was connected to a different cause, such as an adverse medication event.

The exact underlying mechanism of cardiotoxicity remains unclear. One report including two postmortem analyses of cardiac tissues suggested inappropriate proliferation and clonal expansion of T lymphocytes with a high-frequency T-cell receptor targeting an antigen shared by the tumor and striated muscle cells (cardiac and skeletal) [28]. Additionally, in mice, PD-1 has an autoinflammatory role and protects against myocyte damage. Genetic deletion of PD-1 leads to activation of autoantibodies towards troponin I and causes cardiomyopathy [29,30,31]. It is postulated that a similar mechanism can occur in humans with ICIs (by blocking the immune checkpoint receptors); however, postmortem analysis of two patients did not find IgG-autoantibodies in the affected myocardium [28]. Interestingly, a recent study in mice showed potential direct CD8+ mediated toxicity to alfa myosin in cardiomyocytes [32]. Additionally, unlike classical chemotherapeutic agents such as doxorubicin that cause direct cardiac damage, evidence seems to point at an indirect ICI effect on cardiac toxicity through T-cell activation and cytokine hyperproduction, rather than direct damage [33].

Use of dual ICI therapy and in combination with chemotherapy seems to further exacerbate the incidence of cardiac irAEs, showing a possible synergistic effect between these medications and our immune system [34,35]. Additionally, patients with preexisting autoimmune disorders might have on average more cardiac irAEs [36,37]. Both of these findings allude to the possible correlation between immune dysregulation and direct cardiac toxicity. One possible mechanism involves immune tolerance, a process in which our immune system disregards the host cells as a threat. Immune checkpoint inhibitors seem to affect this process within the heart, leaving cardiomyocytes in direct harm’s way of our own immune cells [38].

Certain reporting bias needs to be addressed when discussing cardiotoxic effects of medication, especially possible myocarditis. The recent COVID-19 pandemic and subsequent vaccination have shown association with a surge in myocarditis cases. Both surveillance reporting and systematic reviews have reported such results [39,40]. With this in mind, using the FAERS database to report causality between myocarditis and ICI use in recent years may be somewhat skewed. Another important bias of observational studies is the lack of calculating confounder effect. Myocarditis associated with ICI use has been more commonly reported in patients with history of hypertension, tobacco use, and certain ubiquitous medication such as ACE inhibitors and statins [41]. None of these factors are reported in the FAERS database, limiting the possibility of stratifying the effect one ICI might have had on irAEs compared with another.

In the present study, the most common cardiac AE was myocarditis. It occurred more commonly in men, and all reported cases resulted in serious outcomes, such as hospitalization (30.4%), life-threatening response (14.2%), or death (34.3%). There was no statistical difference between different ICIs and myocarditis. This is consistent with prior data from retrospective studies [7,35,42]. A meta-analysis that included 15 observational studies and compared cardiac AEs between pembrolizumab and nivolumab found that patients treated with pembrolizumab had a lower chance of developing cardiotoxicity (4.6% vs. 7.1%); however, the difference was not statistically significant (*p* = 0.28) [43]. Nonetheless, it is known that myocarditis is more frequent and severe in patients receiving combined immune checkpoint inhibition than monotherapy [28]. We found that reporting of pericardial involvement comparing to other drugs in the database was higher with pembrolizumab (ROR 6.41). Interestingly, reporting of pericardial disease in comparison with other ICIs was higher with pembrolizumab, but we could not find a statistical difference based on disproportionality analysis. The largest retrospective pharmacovigilance study conducted using the World Health Organization’s database for safety reports (VigiBase) had similar results comparing ICIs and all drugs in the database [7]. However, they reported that both myocarditis and pericarditis were more commonly associated with anti-PD1 and anti-PDL1 than anti-CTLA4 therapy [7]. Conversely, we did not observe this difference. As mentioned before, there was no statistical difference in myocarditis/pericardial involvement between different ICIs.

Although myocardial infarction did not show a statistical significance based on the IC value, an interestingly high proportion of AEs was reported for males (71.88%). This gender disbalance may be a result of a number of factors. Firstly, the incidence of myocardial infarction in the general population is greater in the male gender, regardless of cause [44]. This is an important point to make, as the FAERS database only reports the event of myocardial infarction in the presence of pembrolizumab use, not implying a correlation between the two. Thus, in a disease that is more prevalent in the male gender, a higher prevalence of AEs in the gender is to be expected. Secondly, a certain role of sex hormones in the mechanism of action and subsequent effect of immune checkpoint inhibitors has been suggested. Estrogen has been implicated in elevating the immune response, predisposing the female gender to autoimmunity, whilst having a positive effect on overall cardiac mortality [45,46,47]. These factors are just a part of the possible explanations for the variation by gender seen in reporting of cardiac AEs.

Cardiac arrhythmias have previously been reported with ICIs. However, it remains unclear whether this response is due to concurrent cardiotoxicity (myocarditis, heart failure) or due to ICIs themselves. In our analysis, as reported in prior literature, atrial fibrillation was found to be more often reported with pembrolizumab compared with other drugs in the FAERS database. This is similar to a previous FAERS pharmacovigilance study conducted to evaluate cardiac arrhythmia with ICIs [48]. The authors reported statistically significant positive signaling for atrial fibrillation with pembrolizumab (IC025 = 0.19). In addition, they also reported statistically positive signaling of QT prolongation with pembrolizumab (IC025 = 2.0). However, the authors did not report any other disproportionality analysis of statistical measures, and they possibly included combinations of other checkpoint inhibitors with pembrolizumab. One retrospective study that looked at data from 30 patients reported new-onset atrial fibrillation and conduction abnormalities in 30% and 17%, respectively. However, in that study, only 10% of patients were treated with pembrolizumab, while 87% were treated with ipilimumab and/or nivolumab [49]. Interestingly, we also found that atrial fibrillation was higher in men. The mechanism and/or clinical relevance behind this is unknown.

Overall, cardiac AEs were not as frequently reported in relation to ICI use in comparison to other medication. For example, our most frequently reported cardiac AE, myocarditis, was most frequently reported in relation to clozapine use, in 655 cases as monotherapy and 1186 cases as a part of a multidrug regimen (22.47% of all reported myocarditis events for the selected timeframe). In comparison, pembrolizumab was associated with 2.37 times fewer cases (n = 276). As a more staggering comparison, heart failure was most commonly reported with the use of Sacubitril/Valsartan monotherapy, in 3830 cases. Comparing that figure with the 158 cases in pembrolizumab monotherapy, the ICI-related reporting was 24.24 times lower.

The identification and treatment of immune checkpoint inhibitor (ICI)-associated cardiomyopathy largely depends on the availability of dedicated cardio-oncology programs [50]. Fortunately, there has been a notable increase in such programs, both in academic and community settings. Management of these patients typically involves a multidisciplinary approach, with a strong emphasis on collaboration between cardiology and oncology. Both specialties play a critical role in identifying higher-risk patients before the initiation of cardiotoxic cancer treatments and in establishing an appropriate plan for longitudinal follow-up care. The European Society of Cardiology (ESC) guidelines for cancer-related cardiovascular toxicity recommend a patient-centered approach to the timing and frequency of monitoring patients with ICI-associated cardiotoxicity. Toxicity monitoring should be conducted throughout the chemotherapy process. Routine tests include electrocardiograms, echocardiography, troponin levels, and NT-proBNP. Ideally, electrocardiograms should be performed before each chemotherapy cycle, with echocardiography recommended every 2–4 cycles and again 6–12 months after completing treatment. Troponin and NT-proBNP are usually tested at baseline and every 2–4 cycles [5,49,51,52]. Interestingly, no primary prevention strategies have been firmly established. However, general principles for patient care include optimizing lifestyle, smoking and alcohol cessation, and maintaining regular exercise [53]. A recent retrospective analysis by Bhatti and colleagues reviewed 8675 patients exposed to antineoplastic agents who also had type 2 diabetes mellitus and no prior history of cardiomyopathy. These patients were divided into two groups: those on SGLT2 inhibitors and those not. The results showed that patients taking SGLT2 inhibitors had a significantly reduced risk of cancer-related cardiac dysfunction. Furthermore, the SGLT2 inhibitor group demonstrated a statistically significant reduction in heart failure exacerbations, all-cause mortality, all-cause rehospitalization/emergency department visits, and new-onset atrial fibrillation/flutter [54].

Although the FAERS database has many limitations, it plays a critical role in identifying cardiovascular immune-related AEs of immunotherapies, and many different AEs associated with medications used in oncology. One of the most important limitations of the FAERS database is the lack of a denominator (i.e., the total number of patients exposed), therefore it cannot be used to determine true incidence rates of an adverse event [55]. Also, it is very important to emphasize that there can be duplication of the same reports from multiple sources, information can be incomplete, and causality of AEs does not need to be proven before submitting a report [56]. FAERS data have a critical role in treatment decision making in oncology; however, this approach may be insufficient to enable comprehensive interpretation of adverse events for ICIs including analysis of their causality, due to potential influence of concomitant drugs, possible comorbidities, and other patient- or disease-specific factors [56]. More studies are needed to identify important additional clinical and genetic determinants of immune-related cardiovascular AEs and methods to successfully incorporate these data into the FAERS database [9].

## 4. Materials and Methods

### 4.1. Data Source and Extraction Criteria

We conducted an analysis on pembrolizumab adverse events (AEs) based on the FAERS, a publicly available FDA database that contains over 27 million AEs reports, medication error reports, and product quality complaints. These safety reports were submitted to the database by healthcare professionals, patients, and pharmaceutical companies. Reports are managed by the FDA and reviewed by the clinical reviewers in the Center for Biologics Evaluation and Research. The number of individual safety reports is increasing with time and the FAERS database currently contains over 19 million reports. Every report may include, in addition to unique identification numbers, details such as submission and ADR occurrence dates, reporting country, primary source qualifications, patient characteristics (gender, age, weight), suspected and concurrent medications and their uses, ADR descriptions, and their seriousness.

The database was accessed through the interactive FAERS dashboard. The database was queried for anti-PD-1 agent (nivolumab, pembrolizumab, cemiplimab)-, anti-PD-L1 agent (atezolizumab, avelumab, and durvalumab)-, and anti-CTLA-4 agent (ipilimumab)-related AEs, from 1 January 2017 to 31 December 2023. We further filtered the results by the presence of multiple drug regimens per case, selecting only the cases that reported exclusive use of one ICI agent. The next step included filtering the remaining cases by reported adverse event, exporting the data as an Excel table and extracting the demographic data. Duplicate cases were identified through case ID, basic demographic data, and manual read-through of the case list. Reported adverse events were coded based on the preferred term (PT) codes from the Medical Dictionary for Regulatory Activities (MedDRA 21.0) and can be found in the Supplementary Material section. MedDRA^®^ has been developed under the supervision of the International Council for Harmonization of Technical Requirements for Pharmaceuticals for Human Use. The database was accessed on 15 December 2023. We looked for six cardiac-related AEs: “Cardiac Failure”, “Atrial Fibrillation”, “Myocardial Infarction”, “Pericardial Disease”, “Myocarditis”, and “QT Prolongation”. Cardiac failure and congestive cardiac failure were grouped together under the term “Cardiac Failure” for analysis, as were pericarditis and pericardial effusion under the term “Pericardial Disease”.

Data for safety reports were extracted from the publicly available FDA database for each quarter (https://www.fda.gov/drugs/questions-and-answers-fdas-adverse-event-reporting-system-faers/fda-adverse-event-reporting-system-faers-public-dashboard accessed on 5 January 2024). Files from Q1 2017 to Q4 2023 were downloaded and preprocessed. After obtaining the data, AEs resulting from pembrolizumab monotherapy were recorded and included in the disproportionality analysis model. AEs that occurred in combination with pembrolizumab and other ICIs were reported descriptively but were not included in the model.

### 4.2. Data Analysis—Descriptive Analysis

A descriptive analysis of the cardiac-related AEs associated with pembrolizumab was performed to examine the demographic attributes of AEs related to pembrolizumab. Our descriptive analysis included patient characteristics (such as sex and age), type of reaction, and outcome. Outcomes were graded based on severity and included death (grade 5), life-threatening conditions (grade 4), hospitalization (grade 3), disabling outcomes (grade 2), and other outcomes (grade 1). Continuous variables were presented as medians (IQR), while categorical variables were expressed as absolute values (percentages).

### 4.3. Data Analysis—Disproportionality Analysis

To evaluate the possible association between cardiac-specific AEs and pembrolizumab, a disproportionality analysis model was conducted using frequentist methods [calculating the reporting odds ratio (ROR)] and Bayesian methods [calculating the information component (IC)] [57,58,59,60,61]. ROR is a statistical measure used to assess the strength of the association between a drug and a particular adverse event. IC is a measure used to assess and quantify the strength of informativeness between the drug and given adverse events. The formulas of these methods are listed below:

$$ROR=\frac{a/c}{b/d}=\frac{ad}{bc}\;ROR\;95\%\;CI=e^{\ln\left(ROR\right)\pm 1.96\;\sqrt{\left(\frac{1}{a}+\frac{1}{b}+\frac{1}{c}+\frac{1}{d}\right)}}$$

$$Information\;component\;\left(IC\right)={log}_{2}\frac{a+0.5}{a_{exp}+0.5}\;a_{exp}=\frac{\left(a+b\right)*(a+c)}{\left(a+b+c+d\right)}$$

$${IC}_{025}=IC-3.3*{\left(a+0.5\right)}^{-1/2}-2*{\left(a+0.5\right)}^{-3/2}\;{IC}_{975}=IC+2.4*{\left(a+0.5\right)}^{-1/2}-0.5*{\left(a+0.5\right)}^{-3/2}$$

To be statistically significant, the mentioned disproportionately reported signals needed to meet the following criteria [62]:

1. ROR025 (lower limit of the 95% confidence interval of ROR) > 1 and adverse events > 3;
2. IC025 (lower limit of the 95% credibility interval of IC) > 0.

Disproportionally analysis was performed using Microsoft Excel 2021. The incidence of cardiac events of interest was compared between pembrolizumab and other ICIs that are used in monotherapy (cemiplimab, nivolumab, atezolizumab, avelumab, durvalumab, and ipilimumab) and between pembrolizumab and all other drugs in the FAERS database.

## 5. Conclusions

Cardiac AEs caused by pembrolizumab are infrequently reported, more common in men, and are associated with high mortality. The most frequently reported cardiac AEs caused by pembrolizumab are myocarditis, heart failure, atrial fibrillation, and pericardial disease. Further prospective studies are needed to further elucidate the risks of cardiac toxicity in cancer patients and to further evaluate whether these are dose- and gender- dependent.

## 6. Limitations

Our study has important limitations. Like all observational studies, unmeasured confounders could have impacted the results. The main limitation of the study is reflected in the statistical prowess of certain signaling methods. Although not significant in terms of the considered parameters, some results were on the cusp of statistical significance that could have been achieved with a greater sample size. Additionally, the study used one main source of information (the FAERS database), slightly hindering the variety of presented findings; clinically informative parameters such as the event’s time of onset are not reported within this database. Additionally, the FAERS database relies on reporting by volunteers utilizing different resources with significant variability, from healthcare and non-healthcare workers to customers, which may introduce desirability bias. The quality of reported data can vary between one report an another, and not all AEs may be reported.

## Figures and Tables

**Table 1:** Pembrolizumab demographics for 2017–2023.

n (%)		Cardiac Failure	Atrial Fibrillation	Myocardial Infarction	Pericardial Disease	Myocarditis	QT Prolongation
Sex	Female	52 (32.70)	39 (35.14)	23 (23.96)	80 (43.48)	107 (38.77)	0 (0.00)
	Male	101 (63.52)	68 (61.26)	69 (71.88)	104 (56.52)	158 (57.25)	0 (0.00)
	N/A	5 (3.14)	4 (3.60)	4 (4.17)	0 (0.00)	11 (3.99)	0 (0.00)
Age		71.62 ± 9.69 (N/A 37)	71.58 ± 8.05 (N/A 24)	70.00 ± 10.55 (N/A 45)	63.80 ± 13.22 (N/A 43)	69.59 ± 11.55 (N/A 41)	0.00 ± 0.00
Reaction	Serious	158	111	96	184	276	0
	Non-serious	0	0	0	0	0	0
Outcome	Died	67 (42.14)	27 (24.32)	45 (46.88)	32 (17.39)	112 (40.58)	0 (0.00)
	Life-threatening	16 (10.06)	15 (13.51)	9 (9.38)	20 (10.87)	57 (20.65)	0 (0.00)
	Hospitalized	45 (28.30)	35 (31.53)	27 (28.13)	76 (41.30)	68 (24.64)	0 (0.00)
	Disabled	0 (0.00)	0 (0.00)	0 (0.0)	0 (0.0)	1(0.36)	0 (0.00)
	Other	30 (18.87)	34 (30.63)	15 (15.63)	56 (30.43)	38 (13.77)	0 (0.00)

**Table 2:** Disproportionality signal analysis by reporting odds ratio (ROR) and the information component (IC) for pembrolizumab vs. database for 2017–2023. Slash (/): no ROR was calculated since no QT prolongation was reported in the database.

	ROR (95% CI)	IC (97.5% CI)
Cardiac failure	1.69 (1.44–1.97)	0.75 (0.48–1.37)
Atrial fibrillation	1.43 (1.18–1.72)	0.51 (0.19–1.26)
Myocardial infarction	1.22 (1.00–1.49)	0.28 (−0.06–1.09)
Pericardial disease	6.41 (5.54–7.41)	2.64 (2.39–3.22)
Myocarditis	33.87 (29.98–38.26)	4.91 (4.71–5.39)
QT prolongation	/	/

**Table 3:** Disproportionality signal analysis calculated by using the reporting odds ratio (ROR), comparing a monotherapeutical approach using each drug to all other PD-1/PD-L1/CTLA-4 agents, for 2017–2023. Slash (/): no ROR was calculated since no QT prolongation was reported in the database.

Adverse Events	Pembrolizumab	Cemiplimab	Nivolumab	Atezolizumab	Avelumab	Durvalumab	Ipilimumab
Cardiac failure	0.74 (0.63–0.87)	2.05 (0.92–4.61)	1.10 (0.97–1.26)	0.65 (0.48–0.89)	0.81 (0.42–1.56)	0.74 (0.54–1.02)	0.17 (0.07–0.40)
Atrial Fibrillation	0.62 (0.51–0.75)	0.44 (0.06–3.17)	0.80 (0.68–0.95)	0.86 (0.63–1.18)	1.31 (0.72–2.38)	0.52 (0.33–0.79)	0.51 (0.28–0.92)
Myocardial infarction	1.01 (0.81–1.26)	1.52 (0.38–6.10)	1.14 (0.94–1.38)	1.18 (0.82–1.68)	1.41 (0.67–2.97)	1.40 (0.98–1.98)	0.37 (0.15–0.89)
Pericardial disease	1.13 (0.96–1.32)	0.86 (0.21–3.44)	1.14 (0.98–1.32)	1.29 (1.00–1.66)	0.45 (0.17–1.21)	0.72 (0.50–1.04)	0.17 (0.06–0.44)
Myocarditis	1.01 (0.89–1.15)	1.31 (0.54–3.16)	0.85 (0.75–0.97)	0.52 (0.38–0.70)	0.97 (0.57–1.64)	0.53 (0.38–0.74)	0.53 (0.34–0.83)
QT prolongation	/	/	0.31 (0.12–0.76)	/	/	/	1.23 (0.3–5.02)

**Table 4:** Disproportionality signal analysis calculated by using the information component (IC), comparing a monotherapeutical approach using each drug to all other PD-1/PD-L1/CTLA-4 agents, for 2017–2023.

Adverse Events	Pembrolizumab	Cemiplimab	Nivolumab	Atezolizumab	Avelumab	Durvalumab	Ipilimumab
Cardiac failure	−0.37 (−0.63–0.26)	0.91 (−0.50–3.99)	0.12 (−0.08–0.59)	−0.59 (−1.11–0.66)	−0.29 (−1.43–2.26)	−0.41 (−0.94–0.83)	−2.45 (−4.01–0.89)
Atrial Fibrillation	−0.60 (−0.91–0.15)	−0.87 (−4.65–5.33)	−0.26 (−0.53–0.38)	−0.20 (−0.73–1.04)	0.37 (−0.66–2.69)	−0.91 (−1.64–0.79)	−0.92 (−1.95–1.40)
Myocardial infarction	0.02 (−0.32–0.82)	0.46 (−2.14–5.34)	0.15 (−0.14–0.84)	0.22 (−0.37–1.60)	0.45 (−0.85–3.32)	0.45 (−0.13–1.82)	−1.33 (−2.89–2.01)
Pericardial disease	0.14 (−0.10–0.72)	−0.18 (−2.77–4.70)	0.15 (−0.07–0.67)	0.34 (−0.08–1.34)	−1.05 (−2.81–2.64)	−0.45 (−1.06–0.98)	−2.42 (−4.18–1.26)
Myocarditis	0.01 (−0.19–0.49)	0.34 (−1.22–3.68)	−0.19 (−0.38–0.27)	−0.91 (−1.42–0.29)	−0.04 (−0.95–2.03)	−0.87 (−1.42–0.42)	−0.87 (−1.62–0.87)
QT prolongation	−4.77 (−15.10–5.01)	−0.46 (−10.79–9.32)	−1.42 (−2.99–1.92)	−3.15 (−13.47–6.64)	−1.27 (−11.60–8.51)	−2.96 (−13.28–6.83)	0.23 (−2.36–5.12)

## Data Availability

The data used in this analysis is publicly available and can be found at the following link: https://www.fda.gov/drugs/questions-and-answers-fdas-adverse-event-reporting-system-faers/fda-adverse-event-reporting-system-faers-public-dashboard (accessed on 5 January 2024).

## Supplementary Materials

The following supporting information can be downloaded at: https://www.mdpi.com/article/10.3390/ph17101372/s1, The supporting information contains a supplementary table detailing cardiac adverse events grouping as a function of the Medical Dictionary for Regulatory Activities (MedDRA) classification version 21.0; Table S1. Cardiac adverse events grouping as a function of Medical Dictionary for Regulatory Activities (MedDRA) classification version 21.0.
