# Supplementary material for: Pembrolizumab-Associated Cardiotoxicity: A Retrospective Analysis of the FDA Adverse Events Reporting System
Source: Pharmaceuticals (Basel). 2024 Oct 15;17(10):1372. doi: 10.3390/ph17101372 (PMC11510316; doi:10.3390/ph17101372)
Supplement: Supplementary file 1 [file pharmaceuticals-17-01372-s001.zip › pharmaceuticals-3141384-supplementary.pdf]

Supplemental Table S1. Cardiac adverse events grouping as a function of Medical Dictionary for Regulatory Activities (MedDRA) classification version 21.0

Adverse event of interest	Preferred term for adverse event, per MedDRA classification
Cardiac failure	Ischaemic heart failure, Heart failure with reduced ejection fraction, Heart failure, unspecified, Cardiac failure NOS, Cardiac function failure, Insufficiency cardiac, Heart failure with midrange ejection fraction, Decompensated heart failure, Heart failure with preserved ejection fraction, Cardiac failure aggravated, Cardiac insufficiency, Decompensation myocardial, Heart insufficiency, Heart failure (NOS), Failure heart, Recurrent cardiac decompensation, Cardiac failure (NOS), Ischemic heart failure, Heart failure, Decompensation cardiac, Myocardial decompensation, Cardiac function failed
Cardiac failure congestive	Rheumatic heart failure (congestive), Heart failure, congestive; Cardiac failure, congestive; Congestive heart failure with acute exacerbation, Congestive heart failure, Failure heart congestive, Bi-ventricular failure, Congestive cardiac failure aggravated, Congestive cardiac failure
Atrial fibrillation	Atrial fibrillation with slow ventricular response, Paroxysmal atrial fibrillation, Fibrillation atrial aggravated, Permanent atrial fibrillation, Chronic atrial fibrillation, Alcoholic atrial fibrillation, Fibrillation paroxysmal atrial, Atrial fibrillation aggravated, Bradyarrhythmia absoluta, Tachyarrhythmia absoluta, Asymptomatic atrial fibrillation, AFib, Atrial fibrillation with rapid ventricular response, Auricular fibrillation, Arrhythmia absoluta, Recurrent symptomatic atrial fibrillation, Fibrillation atrial, Recurrent atrial fibrillation, Persistent atrial fibrillation, Atrial fibrillation paroxysmal
Electrocardiogram QT prolonged	Electrocardiogram QT prolonged
Myocardial infarction	Transmural myocardial infarction, Posterolateral myocardial infarction, Inferolateral myocardial infarction, Age indeterminate inferolateral myocardial infarction, Cardiopathy necrotic, Small focal myocardial infarction, Inferior

	myocardial infarction, Non-Q wave MI, Apical myocardial infarction, Heart attack, Posterior myocardial infarction, Attack heart (NOS), Q wave MI, Age indeterminate posterior myocardial infarction, Septal myocardial infarction, Age indeterminate lateral myocardial infarction, Myocardial infarction old, Inferior MI, Age indeterminate myocardial infarction, Post MI, Right ventricular infarction, Age indeterminate posterolateral myocardial infarction, Anteroseptal necrosis, Anterior myocardial infarction, Age indeterminate anterior myocardial infarction, Myocardial reinfarction, Subendocardial myocardial infarction, Age indeterminate anterolateral myocardial infarction, Anterolateral myocardial infarction, Anteroseptal infarction, Myocardial infarct, Age indeterminate inferior myocardial infarction, Age indeterminate anteroseptal myocardial infarction, Myocardial infarction old healed, Age indeterminate septal myocardial infarction, Infarct myocardial, Old myocardial infarction, Anterior MI, Age indeterminate right ventricular infarction, Age indeterminate inferoposterior myocardial infarction, Lateral myocardial infarction, Posteroinferior myocardial infarction
Pericardial effusion	Pericardial fluid exudate, Effusion pericardial bloody, Hydropericardium, Effusion pericardial
Pericarditis	Relapsing pericarditis, Pericarditis sicca, Pericarditis epistenocardiaca, Recurrent pericarditis, Acute idiopathic pericarditis, Hydropericarditis, Acute pericarditis, unspecified, Subacute pericarditis, Exudative pericarditis, Pericarditis NOS, Acute pericarditis
Myocarditis	Myocarditis, unspecified, Toxic myocarditis, Myocarditis NOS, Lymphoid interstitial myocarditis, Acute myocarditis, unspecified, Asymptomatic myocarditis, Subclinical myocarditis, Myocarditis interstitial, Idiopathic myocarditis, Acute myocarditis, Focal myocarditis
